# Efficacy of a Mobile Social Networking Intervention in Promoting Physical Activity: Quasi-Experimental Study

**DOI:** 10.2196/12181

**Published:** 2019-03-28

**Authors:** Huong Ly Tong, Enrico Coiera, William Tong, Ying Wang, Juan C Quiroz, Paige Martin, Liliana Laranjo

**Affiliations:** 1 Centre for Health Informatics Australian Institute of Health Innovation Macquarie University Sydney Australia

**Keywords:** mobile apps, fitness trackers, exercise, social networking

## Abstract

**Background:**

Technological interventions such as mobile apps, Web-based social networks, and wearable trackers have the potential to influence physical activity; yet, only a few studies have examined the efficacy of an intervention bundle combining these different technologies.

**Objective:**

This study aimed to pilot test an intervention composed of a social networking mobile app, connected with a wearable tracker, and investigate its efficacy in improving physical activity, as well as explore participant engagement and the usability of the app.

**Methods:**

This was a pre-post quasi-experimental study with 1 arm, where participants were subjected to the intervention for a 6-month period. The primary outcome measure was the difference in daily step count between baseline and 6 months. Secondary outcome measures included engagement with the intervention and system usability. Descriptive and inferential statistical tests were conducted; posthoc subgroup analyses were carried out for participants with different levels of steps at baseline, app usage, and social features usage.

**Results:**

A total of 55 participants were enrolled in the study; the mean age was 23.6 years and 28 (51%) were female. There was a nonstatistically significant increase in the average daily step count between baseline and 6 months (mean change=14.5 steps/day, *P*=.98, 95% CI –1136.5 to 1107.5). Subgroup analysis comparing the higher and lower physical activity groups at baseline showed that the latter had a statistically significantly higher increase in their daily step count (group difference in mean change from baseline to 6 months=3025 steps per day, *P*=.008, 95% CI 837.9-5211.8). At 6 months, the retention rate was 82% (45/55); app usage decreased over time. The mean system usability score was 60.1 (SD 19.2).

**Conclusions:**

This study showed the preliminary efficacy of a mobile social networking intervention, integrated with a wearable tracker to promote physical activity, particularly for less physically active subgroups of the population. Future research should explore how to address challenges faced by physically inactive people to provide tailored advices. In addition, users’ perspectives should be explored to shed light on factors that might influence their engagement with the intervention.

## Introduction

### Background

There is strong evidence of the effectiveness of regular physical activity in the prevention of several chronic diseases and associated premature death [1,2]. Furthermore, there appears to be a dose-response relationship between physical activity and health status [3,4]. Yet, despite the importance of physical activity, 27.5% of adults worldwide are insufficiently active [5], highlighting the need for interventions to promote physical activity.

Behavioral informatics interventions (ie, using health information technology to facilitate behavior change) have become increasingly popular in recent years [6]. A key element to behavior change success is the use of behavior change theories, models and techniques to better understand the causal mechanisms and influencing factors of the behavior, and the context of the intervention [6]. In addition, in recent years, researchers have encouraged intervention developers to describe their interventions in terms of the specific behavior change techniques [7]. A behavior change technique is an *observable, replicable, and irreducible component* of an intervention, intended to alter causal processes that regulate behavior [7]. Behavior change techniques can be linked to existing theories and models, and they provide a more transparent, replicable approach to the design and evaluation of behavior change interventions [7,8].

To date, several behavior change theories and models have indicated the importance of the link between social factors and health-related behaviors [9-11]. In particular, researchers have demonstrated that existing networks of friends and family exert great influence on individual health behavior [12,13], suggesting the potential of leveraging social networks to deliver physical activity interventions [14]. Social networks refer to the webs of an individual’s relationships, which give rise to various functions such as social influence, social companionship, social support, and social comparison [15]. To date, several studies have found strong evidence that behavior change techniques such as social support and social comparison increase physical activity levels [16-18]. Though these interventions seem promising, their potential can be missed when they are not easily disseminated or accessible to a large audience [19]. A potentially useful way to disseminate social network interventions for physical activity is through the use of Web-based social networks. Web-based social networks, which are now ubiquitous in our lives, allow users to create a personal profile and connect with other users [20]. Several meta-analyses have found that online social networks can have positive, significant effects on behavior change [21,22].

In addition to social aspects, many studies have also highlighted the importance of other behavior change techniques, such as self-monitoring or goal setting, in physical activity [23,24]. Mobile health (mHealth) technologies such as mobile apps and wearable trackers offer new opportunities to deliver these behavior change techniques. In particular, recent mHealth technologies can reach individuals continuously, allowing users to self-monitor their physical activity [25] and providing real-time feedback [26]. mHealth interventions have increasingly been used in physical activity interventions, reporting significant, moderate improvements in step counts [27-29]. Given their potential, interventions combining mHealth technologies and online social networks might be particularly effective in promoting physical activity.

To date, researchers have largely examined the effects of mHealth and Web-based social networks on physical activity in isolation [30-37]. There are a few studies that evaluated the feasibility and effectiveness of interventions with both mHealth and Web-based social network components, showing user acceptability and moderate increases in physical activity levels [38-42]. However, these studies often examine online social networks as an additional feature (eg, a Facebook group), not integrated within a mobile app. In addition, it is also essential to examine usage metrics and usability determinants of mHealth interventions, as these factors reflect true user engagement and can largely influence the effects of the intervention [43].

### Objectives

The aim of this study was to pilot test a social networking mobile app, connected with a wearable tracker to promote physical activity. In particular, we investigated (1) the intervention efficacy on physical activity and (2) participant engagement and usability of the intervention. The secondary aims were to explore the effects of social features on physical activity levels and the association between engagement with the mobile app and physical activity levels.

## Methods

### Study Design

This study is part of a larger mixed-methods feasibility study on the use of a social networking mobile app to promote physical activity and weight management [19]. In particular, this paper reports on the quantitative results related to the physical activity outcomes of a pre-post, 1-arm quasi-experiment where participants were subjected to the intervention for a 6-month period. Results related to weight outcomes of the study will be reported in a forthcoming publication. The design and conduct adhered to the Consolidated Standards of Reporting Trials 2010 statement—extension to randomized pilot and feasibility trials, where applicable [44].

### Ethics

Ethics approval was granted by Macquarie University’s Human Research Ethics Committee for Medical Sciences (ethics reference number 5201600716).

### Study Settings and Participants

A total of 55 participants (mean age 23.6 years, 51% [28/55] female), mostly Macquarie University students and staff (Sydney, Australia), were recruited using purposive sampling techniques. Given the nature of this study, the sample size was pragmatically chosen to enable a comprehensive assessment of the feasibility of the intervention before conducting a randomized controlled trial (RCT) [44]. Recruitment channels included posters around university campus, website information, and Facebook. Eligible participants were healthy adults with sufficient knowledge of English to understand and participate in the study, they had planned to be living in Sydney for the duration of the study and owned a mobile phone (iOS or Android) with internet access. Exclusion criteria were pregnancy, body mass index (BMI) below 17, earlier history of eating disorders, or having diabetes or other comorbid conditions that could impact study participation (eg, severe mental illness, end-stage disease). Participants were screened for eligibility via an online questionnaire.

Eligible participants were invited to attend the initial study session at the research center, where they received information about the purpose of the study and signed the consent form. Subsequently, participants filled in a questionnaire about their demographic characteristics and smartphone usage (eg, type of smartphone used, hours per day using the smartphone), and their baseline measurements (ie, weight, height) were assessed. At the end of the study, participants were invited to attend a postintervention session in which they completed the System Usability Scale (SUS) survey [45], and their weight was measured again.

### Intervention Description

The intervention bundle involved 3 components, including a mobile app (named fit.healthy.me), a wearable tracker, and short message service (SMS) text messages and emails. In particular, the fit.healthy.me app was developed on the basis of several behavior change techniques, such as self-monitoring of physical activity, social support, and social comparison. In the app, the social features were composed of *My team*, *Social forum*, and *Private messages*. *My team* allowed participants to visualize and compare their step counts with others and *follow* other people, whereas *Social forum* and *Private messages* allowed participants to interact and provide social support to each other.

To enable the automation of self-monitoring, the fit.healthy.me app was integrated with the Fitbit Flex 2 wearable tracker [19]. In particular, the Fitbit Flex 2 was wirelessly synced with fit.healthy.me (via the Fitbit app programming interface). Fitbit Flex 2 uses accelerometer technology to measure acceleration signals, which are then converted to step count—a common indicator of physical activity. Research has demonstrated good reliability and validity in using Fitbit Flex 2 for measuring step count in free-living conditions [46,47].

In addition, prompts and cues (ie, SMS text messages and emails) were sent every 2 weeks to remind users to wear the fitness tracker during waking hours and check fit.healthy.me at least once every day. A detailed description of the modes of delivery and features of the intervention is presented in Table 1. Screenshots of the mobile app are provided in Multimedia Appendix 1.

Before the study commencement, the fit.healthy.me app underwent development testing [48] within the research center. Participants were provided access to the intervention by downloading the app from the Apple app Store or Google Play. During the study, participants could email or call the study team if they required any technical assistance. A research team member with clinical expertise also regularly monitored the study and responded to any concerns raised by participants. As an incentive for participation in the study, individuals were offered to keep the tracker at the end of the 6-month period.

### Measures

This paper specifically reports on 3 aspects of the study results: (1) the efficacy of the intervention on physical activity measures, (2) participant engagement with the intervention, and (3) the usability of the fit.healthy.me app.

#### Efficacy in Promoting Physical Activity

The primary outcome measure for this study was the difference in the daily step count between baseline and 6 months, which was measured using the Fitbit Flex 2 and retrieved via the Fitbit app programming interface. To enable the collection of baseline daily step count, participants underwent a 7-day period after the initial study session where they were not able to log in to fit.healthy.me but were asked to use the Fitbit Flex 2 every day; the baseline measure was obtained by averaging the number of steps per day the first 7 days. The final step count was determined by computing the average number of steps per day on the last week where participants had at least four valid days [49]. A valid day of step count was defined as at least 10 hours of wear time during that day (Table 2) [47]. The wear time was calculated by subtracting nonwear time from 24 hours; nonwear time was defined if no step counts were detected over a period of at least 60 continuous min, allowing for 2 min of counts between 0 and 100 [49,50].

Posthoc subgroup analysis was carried out for participants with different physical activity levels at baseline (≥10,000 steps per day vs <10,000 steps per day). A total of 10,000 steps per day were used as a threshold, as this goal is acknowledged as a reasonable target for healthy adults [51-53].

#### Participant Engagement

Participant engagement with the intervention was assessed using multiple measures (Table 2). In particular, retention was defined as attendance at the 6-month final session. Participants who came to the final sessions were considered *completers* and participants who did not come were considered to have dropped out of the study. For the Fitbit Flex 2, engagement was measured by the mean number of days a valid step count was logged (participants were considered to have a valid step count if they wore the Fitbit for at least ten hours on any given day). For the fit.healthy.me app, engagement was measured by both the length of usage (ie, the mean number of days of usage) and frequency of usage (ie, the number of times participants used the app and each feature). A participant was considered to have used the app in a day if he or she used any features of the app at any time of that day. Similarly, a participant was considered to have used a social feature if he or she clicked on any of *My team*, *Social forum*, and *Private messages* features at any time. Every time a participant used an app feature, the timestamp and the name of that feature were automatically saved into our local database. These data were summarized to show participant engagement with the fit.healthy.me app at the end of the study.

**Table 1:** Intervention features and behavior change techniques.

Modes of delivery	Features	Behavior change techniques^a^
fit.healthy.me app	My measures	Self-monitoring of behavior (ie, number of steps per day)
	My team	Social comparison
	Social forum	Social support (emotional); Social comparison
	Private messages	Social support (emotional); Social comparison
	My journey	Instruction on how to perform the behavior
Fitbit Flex 2	Fitness wearable tracker	Self-monitoring of behavior (ie, physical activity)
SMS^b^ text messages and emails	Reminders	Prompts/cues

^a^Classified according to the behavior change techniques taxonomy developed by Michie et al [7].

^b^SMS: short message service.

**Table 2:** Definition and calculation of engagement measures.

Engagement measures		Definition
**Retention^a^**		
	Completers	Participants who came to the final sessions
	Noncompleters	Participants who did not come to the final sessions (dropout attrition)
	Retention rate	Percentage of completers out of all 55 participants
**Fit.healthy.me app usage**		
	Length of usage	The mean number of days of usage
	Frequency of usage	The mean number of times participants used the app and each feature
	Nonusage attrition	Participants who did not use the app at all in the last month of the study
**Fitbit Flex 2 tracker usage**		
	Length of usage	The mean number of days a valid step count was logged
	A valid day of step count	Having at least ten hours of wear time
	Wear time	Calculated by subtracting nonwear time from 24 hours
	Nonwear time	Defined if no step counts were detected over a period of at least sixty continuous minutes, allowing for 2 min of counts between 0 and 100 [49,50]

^a^Adapted from Eysenbach (2005) [43].

#### Usability

Participants completed the SUS [45] to assess the usability of the fit.healthy.me app. The SUS is a validated questionnaire comprising a standard set of 10 statements that seek users’ opinions on the usability of a system [45]. SUS has been widely used to evaluate usability within commercial and research studies (including mobile apps) for over 30 years [54-56]. Participants were asked to rank the statements on a 5-point Likert scale from strongly disagree (scored as 1) to strongly agree (scored as 5). Final scores of the SUS can range from 0 to 100, with higher scores indicating better usability [57]. A study collecting 10-years’ worth of SUS data from over 200 studies found that the average score is around 70, suggesting that a SUS score of 70 might be considered acceptable [57]. A list of the statements and explanation for calculation of the SUS scores is provided in Multimedia Appendix 2.

#### Statistical Analysis

Participants’ demographic characteristics, intervention usage data, and engagement metrics were analyzed descriptively using means, SD, and frequency counts. Wilcoxon signed-rank test was used to determine whether the number of days participants used the fit.healthy.me app differed between the first and last (sixth) month of the study. SUS score was calculated to determine the usability of the fit.healthy.me app [45].

To investigate the efficacy of the intervention, the difference between average step count at baseline and final weeks was assessed using a paired, 2-tailed *t* test. A total of 3 participants did not have valid data for at least four days at the end of the study, and thus they were excluded from the analysis. Kendall tau-b test was used to measure the correlation between total engagement with the fit.healthy.me app and changes in daily step count.

Posthoc subgroup analyses were carried out for participants with different levels of steps at baseline, app usage, and social features usage. As mentioned above, in terms of physical activity, 10,000 steps per day were used as a cut-off point to define high- versus low-level physical activity [51-53]. In terms of app usage and social features usage, the median was used as a cut-off point to determine frequent versus nonfrequent usage. Independent 2-sample *t* tests were used for normally distributed numerical data; for nonnormal data, the Wilcoxon rank-sum test was used. Chi-square tests were used for categorical data. For statistically significant results, effect sizes (ie, Cohen *d*) were calculated [58].

Data were analyzed using R version 3.5.0 (R Foundation for Statistical Computing) [59-63]. The significance level for all statistical tests was set at *P*<.05, 2-tailed, and 95% CIs were calculated where applicable.

## Results

### Participant Flow and Recruitment

Recruitment occurred from April to May 2017. A total of 423 people completed an online questionnaire to assess their eligibility; 55 of them met the eligibility criteria, consented to participate, and attended the preintervention session. The most common reasons for ineligibility were pregnancy and chronic diseases. After each participant completed the 6-month period, they were sent an automatic email, inviting them back for the final sessions. Out of 55 initial participants, 45 participants returned for the final session (ie, completers). Step data were collected for all 55 participants during the 6-month intervention period. Given our definition of valid days and the condition that at least four valid days were needed to compute the weekly average, not all participants had the final step count in week 26 (median final week number: 21; interquartile range: 10-25).

### Sample Characteristics

A summary of the differences in baseline characteristics between enrolled participants and completers is presented in Table 3. At baseline, participants had a mean age of 23.6 years (SD 4.6). Furthermore, 28 (51%) were female, and 42 (76%) were university students. The average BMI was 26.5 kg/m^2^ (SD 6.8), with nearly half of the participants (24/55, 44%) in the normal weight range. Participants reported using a smartphone for 5.6 hours (SD 3.4) per day, on average; most users (36/55, 66%) had an iPhone. The majority of participants (49/55, 89%) said that the most used apps in their phones were social media apps, whereas 10% (6/55) said fitness apps. There were no statistically significant differences between enrolled participants and completers.

### Physical Activity Measures

**Figure 1:**
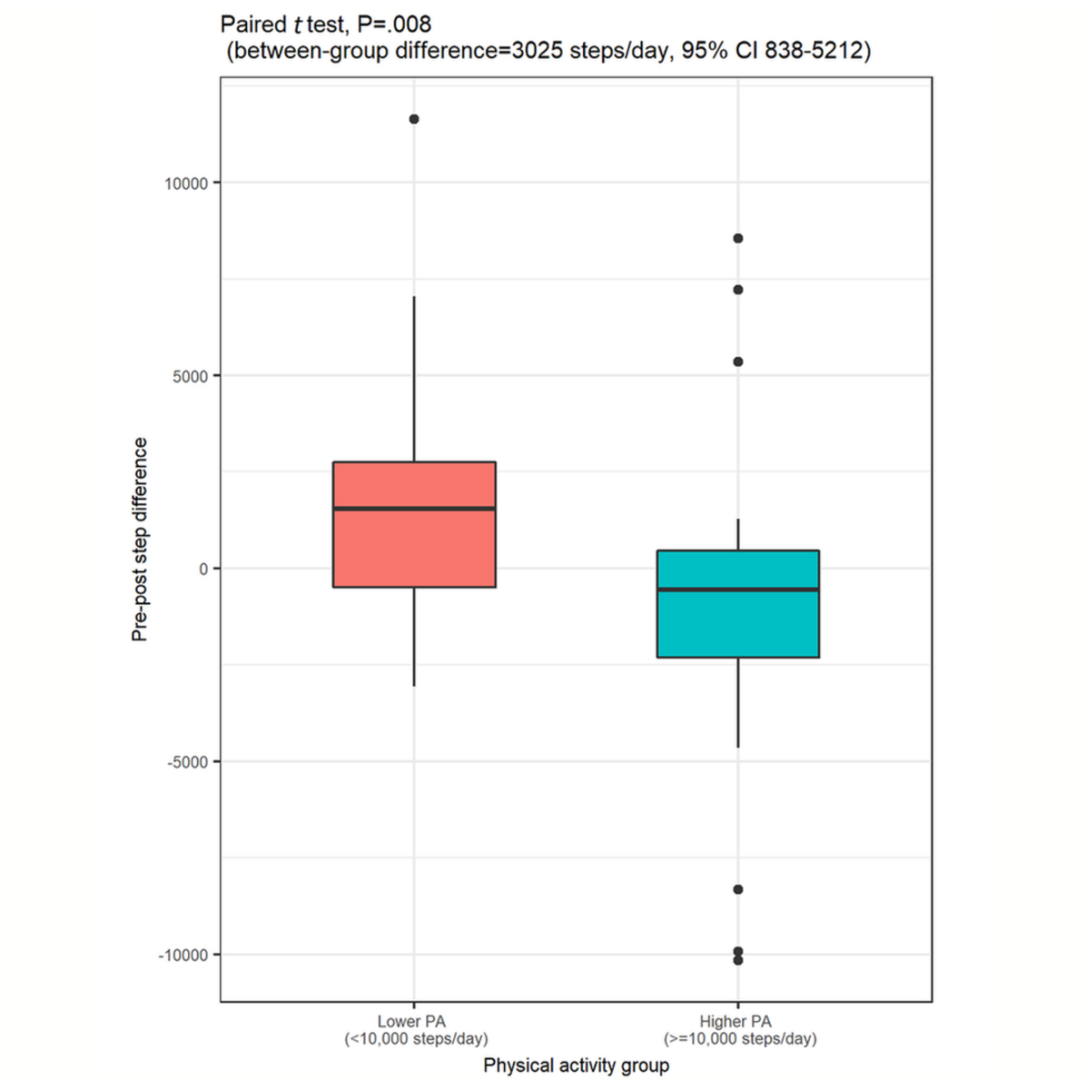
Boxplots of the differences in pre-post daily step count between the lower and higher physical activity groups. PA: physical activity.

On average, daily step count did not change between baseline and 6 months (mean difference=14.5, *P*=.98, 95% CI –1136.5 to 1107.5). A subgroup analysis comparing the higher physical activity group with the lower physical activity group (at baseline) showed that the lower physical activity group experienced a statistically significant increase of 3025 steps in daily step count between baseline and post intervention (*P*=.008, 95% CI 837.9-5211.8], Cohen *d*=0.80; Table 4 and Figure 1). Multimedia Appendix 3 shows box plots for participants’ daily step count at each week of the study. There were no statistically significant changes in average daily step count between different levels of app usage (*P*=.42; Multimedia Appendix 4) or different levels of social feature usage (*P*=.25; Multimedia Appendix 5). Total engagement with the fit.healthy.me was not directly associated with change in daily step counts (Kendall tau-b=–0.11, *P*=.25).

**Table 3:** Differences in baseline characteristics between enrolled participants and completers.

Measures		Enrolled participants (n=55)	Study completers (n=45)	*P* value
Age, mean (SD)		23.6 (4.6)	24.2 (4.7)	.51^a^
Female, n (%)		28 (51)	22 (50)	.52^b^
Weight (kg), mean (SD)		78.1 (22.3)	77.8 (21.2)	.99^a^
Body mass index (kg/m^2^), mean (SD)		26.6 (6.8)	26.7 (6.5)	.94^a^
**Body mass index categories^c^, n (%)**				
	18-18.49	3 (6)	1 (2)	.14^b^
	18.5-24.99	24 (44)	22 (49)	.19^b^
	25-29.99	15 (27)	10 (22)	.16^b^
	≥30	13 (24)	12 (27)	.48^b^
Steps/day, mean (SD)		10,967.2 (3907.4)	10,896.3 (4206.2)	.93^a^

^a^Assessed using 2-sample *t* tests.

^b^Assessed using chi-square tests.

^c^According to the World Health Organization, a body mass index of less than 18.5 is classified as underweight, 18.5-24.9 is normal, 25-29.9 is preobese, and ≥30 is obese [64].

**Table 4:** Differences in characteristics between lower and higher physical activity subgroups at baseline.

Measures	<10,000 steps/day (n>=20), mean (SD)	≥10,000 steps/day (n=35), mean (SD)	*P* value (95% CI)
Baseline weight (kg)	77.0 (26.3)	78.6 (20.1)	.80^a^ (–14.3 to 11.0)
Baseline body mass index (kg/m^2^)	26.4 (7.8)	26.6 (6.2)	.91^a^ (–4.1 to 3.6)
Duration of app usage (days)	16.1 (15.3)	15.4 (17.0)	.51^b^ (–4.0 to 7.0)
Intensity of app usage (times)	1487.0 (1244.7)	1719.1 (1561.6)	.79^b^ (–559 to 860)
Pre-post intervention step difference	1992.3 (3598.3)	–1032.6 (3894.7)	.008^c^ (837.9-5211.8)

^a^Assessed using 2-sample *t* test.

^b^Assessed using Wilcoxon rank-sum test.

^c^denotes statistical significance.

### Participant Retention and Engagement

**Figure 2:**
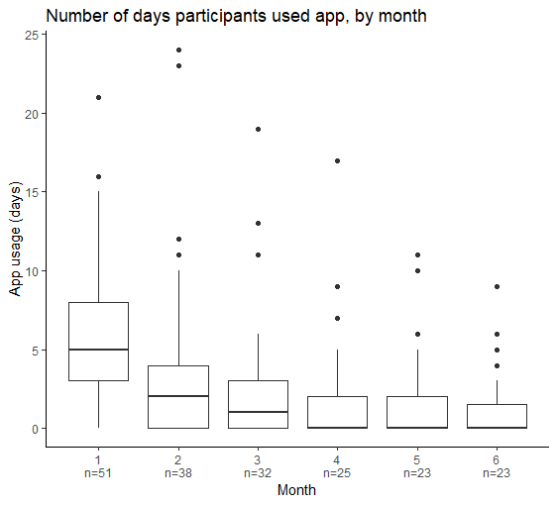
Boxplots of the number of days participants used the fit.healthy.me app, by month.

The retention rate was 82%. Overall, the length of usage of the Fitbit Flex 2 tracker was higher than app and social features. *My Team* and *My Measures* had a higher level of engagement compared with *Social Forum* and *Private Messages* (Table 5). In general, app usage decreased over time (Figure 2). In particular, the number of days participants used the app in the last month of the study significantly decreased from the first month of the study (*P*<.001, 95% CI –5.5 to –4). In total, 4 participants did not use the app at all throughout the study. Subgroup analyses showed that there were no statistically significant differences in any characteristics between frequent and nonfrequent app users (Multimedia Appendix 4).

### System Usability Scale

Out of 55 participants, only 45 returned to the postintervention sessions and completed the SUS. The mean SUS score was 60.1 (SD 19.2). Two-third of the participants (N=30) gave a SUS score lower than 70, indicating low usability [57]. Furthermore, 7 participants rated the app’s usability as moderate and 8 participants rated it as having high usability. Multimedia Appendix 2 presents responses to individual SUS statements. Posthoc subgroup analysis indicated that frequent app users gave a higher SUS score than nonfrequent users (*P*=.04, 95% CI 0.6-25.3; Multimedia Appendix 4).

**Table 5:** Length and frequency of usage of the Fitbit Flex 2, fit.healthy.me app, and social features. Study duration was 183 days.

Engagement measures and usage data			Mean (SD)	Range
**Fitbit Flex 2 usage**				
	Days valid step count were logged via Fitbit (days)		66 (48.7)	5-183
**App usage**				
	Length (days)		15.7 (16.2)	0-63
	Frequency (times)		1634.7 (1446.8)	0-6317
**App features usage**				
	**Frequency (times)**			
		My measures	44.2 (47.8)	0-228
		My team^a^	59.0 (51.6)	0-203
		Social forum^a^	21.8 (37.5)	0-213
		Private messages^a^	9.2 (20.8)	0-88
		My journey	17.0 (13.0)	0-63

^a^Social features included *My team*, *Social forum*, and *Private messages*.

## Discussion

### Principal Findings

There was a nonstatistically significant increase in the average daily step count between baseline and 6 months. Subgroup analysis comparing the higher and lower physical activity groups at baseline showed that the latter experienced a statistically significant increase in average daily step count between baseline and postintervention, suggesting the app might be more beneficial for specific subgroups of the population (eg, less physically inactive individuals). At 6 months, the retention rate was 82%; 42% participants used the fit.healthy.me app at least once during the last month of the study.

To the best of our knowledge, our study is the first to evaluate a mobile social networking intervention integrated with a wearable tracker. Other studies have examined interventions composed of either mobile technologies [30-33] or online social networks [34-37] in isolation, and thus evidence on the efficacy and feasibility of an intervention combining both was limited until now. Even though several studies have incorporated social features in mHealth interventions, these features were often included as an additional component (eg, Facebook group) rather than being fully integrated with the mobile app [38,39,41,42,65,66].

### Efficacy in Promoting Physical Activity

Our study found that compared with the higher physical activity group, the lower physical activity group at baseline experienced a significant increase of 3025 steps in daily step count, suggesting that specific populations (eg, less physically active people) might benefit more from the use of a mobile social networking app. Earlier research has outlined the importance of considering particular challenges and barriers that inactive people might face when designing fitness technology. For example, several studies have suggested that although self-regulation techniques (ie, goal setting, self-monitoring, and feedback on behavior) and social support are often present in fitness technology, other behavior change techniques such as action planning or environment restructuring are present less often and might be particularly useful for inactive people [67,68]. It is worth noting that even increases of 2000 steps per day are associated with reduced risk of cardiovascular disease, given the dose-response relationship between physical activity levels and health benefits [69]. Altogether, the use of behavioral informatics such as ours seem promising, and it should be confirmed by fully powered RCTs.

### User Retention, Engagement, and Usability

The retention rate of our study was 82%, which is consistent with the reported retention rates of around 70% to 90% in other mHealth and online social networks interventions [21,38,39,70-72]. Our study also revealed that app usage declined over time—a phenomenon frequently observed in other apps for physical activity [29,73,74]. It is known that initially, users tend to be attracted to new technologies; over time, disengagement can be triggered by either internal factors such as lack of time, or it can be triggered by external factors such as usability issues and technological problems [75]. A possible explanation for the decline in usage of our app could be usability issues. In fact, two-third of our users gave a SUS score of lower than 70 to the fit.healthy.me app, indicating low usability [57]; nonfrequent users were more likely to give a lower SUS score. Indeed, when a user experiences a usability flaw, the negative experience might outweigh other positive features of the technology (a phenomenon known as *negativity bias*) [76], and it can subsequently lead to lower engagement. The link between usability and engagement has been frequently demonstrated in previous research [75]. Of note, the technology acceptance model highlights the importance of perceived usefulness and perceived ease of use (concepts overlapping with many aspects of usability) [45,77-79] in users’ acceptance and adoption of technology [80,81]. Hence, it is important to address usability to maximize user engagement.

We also found that usage levels varied among different features. In particular, *My team* attracted a significantly higher level of engagement compared with *Social forum* and *Private messages*. This difference could possibly be because of the format and content presented in each feature: *My team* supports social comparison via displaying summary statistics and graphs, whereas the *Social forum* and *Private messages* features support discussion among users. It can be hypothesized that users found more utility in the numerical and graphical social comparison aspects of *My team* to the discussion-based nature of other social features, suggesting the need to explore how to effectively deliver social behavior change techniques to maximize engagement.

### Strengths and Limitations

This study has several strengths. First, we assessed a range of features supporting different behavior change techniques to examine the individual aspects of this multicomponent intervention. Second, we reported different measures of engagement, including retention rate, nonusage attrition, and engagement metrics with different intervention components to shed light on the attrition problems in behavioral informatics interventions [43,82]. Finally, the intervention was fully integrated with wireless tracking devices, and thus the wireless tracking devices eliminated the reliance on self-reported data.

The findings of this study must be interpreted in light of some limitations. Given that this was a quasi-experimental study with a single-arm pre-post design, we cannot infer causation from our results. Possible confounders might have been at play, and thus the results should be interpreted with caution. Moreover, we had a purposely small and homogenous sample, which affects generalizability of the study. Another limitation is related to the handling of missing data in daily step count. Due to our definition of valid days of step count and the condition that participants needed to have at least four valid days of daily step count within a week to compute the weekly average, not all participants had the final step count in the last month of the study; therefore, we calculated the final step count on the basis of the last week where participants had at least four valid days. Although this method allowed us to include more participants in the analysis (and thus avoid selection bias resulting from excluding participants from the analysis), it can potentially bias the results in other ways (eg, overestimation of the final step count in case the daily step count decreases over the study duration). In addition, as the fit.healthy.me app was developed for research purposes, it lacked the advanced features and design aspects that would be available in commercially available fitness apps. Usability testing was assessed using the SUS and not done extensively. All posthoc subgroup analyses were exploratory and might be subject to type I error. In particular, in our analysis comparing different physical activity subgroups, our focus was on the difference between baseline and final weeks, and the analysis did not take into account all 26 weeks (Multimedia Appendix 3). Future work exploring the time series nature of physical activity data and analyzing and modeling weekly trends might reveal more in-depth information about users’ behavioral patterns and provide more robust results. Finally, in this study, we only used step count as a measure of physical activity. Future research might consider other measures, such as intensity of physical activity (light, moderate, and vigorous) or sedentary time [83,84].

### Implications

This study highlights several important implications regarding the design and implementation of behavioral informatics interventions for physical activity. First, our findings suggest that wearable devices and mobile social networking apps can work in synergy to facilitate behavior change, particularly in physically inactive groups. In particular, wearable trackers can automate self-monitoring—an important task in behavior change [23,85], whereas mobile apps can provide a platform to support other relevant behavior change techniques, such as providing feedback on behavior, goal setting, or social comparison [86]. Several studies have also suggested that social interaction can enhance engagement [28,87], highlighting the potential of integrating social features in technological interventions.

Furthermore, it is important to note that physically inactive groups might face additional challenges, and thus future research should also consider the potential of other behavior change techniques in these interventions. Perhaps fitness technology could prompt individuals to identify the particular barriers they face regarding physical activity [67], and it could facilitate the tailoring of specific recommendations accordingly. Tailored advices can be more helpful and relevant to users [88,89], potentially leading to more effective interventions in this subgroup of the population. In addition, future research should also explore users’ preferences and perspectives on factors that might influence their engagement, to maximize the effectiveness of mHealth interventions in promoting physical activity.

### Conclusions

Our study showed preliminary evidence that mobile social networking interventions, integrated with wearable trackers, can help to promote physical activity. Future research needs to explore how to best support barriers faced by physically inactive people and accordingly provide tailored recommendations to maximize intervention effectiveness.

## Appendix

Multimedia Appendix 1 Screenshots of fit.healthy.me app.

Multimedia Appendix 2 Responses to individual System Usability Scale statements.

Multimedia Appendix 3 Boxplots of the 55 participants' daily step count over 26 study weeks.

Multimedia Appendix 4 Differences in characteristics between frequent app users and nonfrequent app users.

Multimedia Appendix 5 Differences in characteristics between frequent users and nonfrequent users of the social features in the fit.healthy.me app.

## Abbreviations

BMI: body mass index
mHealth: mobile health
RCT: randomized controlled trial
SUS: System Usability Scale
