# Supplementary material for: Efficacy of a Mobile Social Networking Intervention in Promoting Physical Activity: Quasi-Experimental Study
Source: JMIR Mhealth Uhealth. 2019 Mar 28;7(3):e12181. doi: 10.2196/12181 (PMC6458538; doi:10.2196/12181)
Supplement: Multimedia Appendix 1 [file mhealth_v7i3e12181_app1.pdf]

Appendix 1: Screenshots of fit.healthy.me app

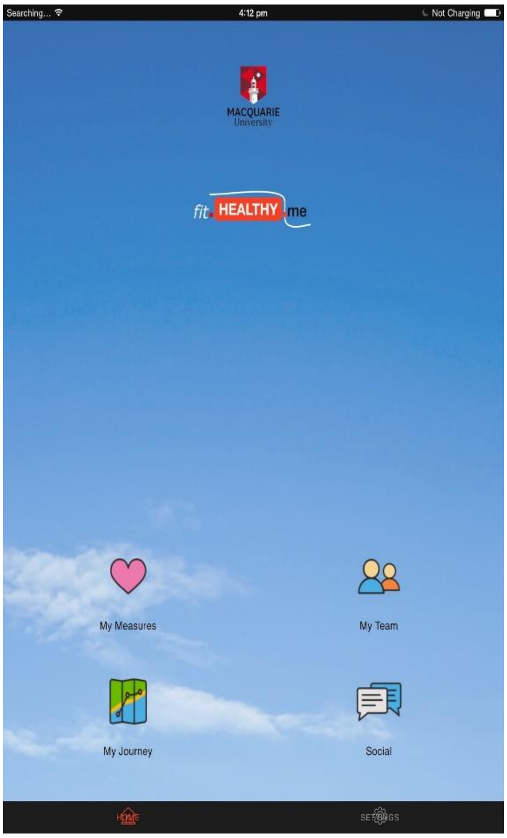

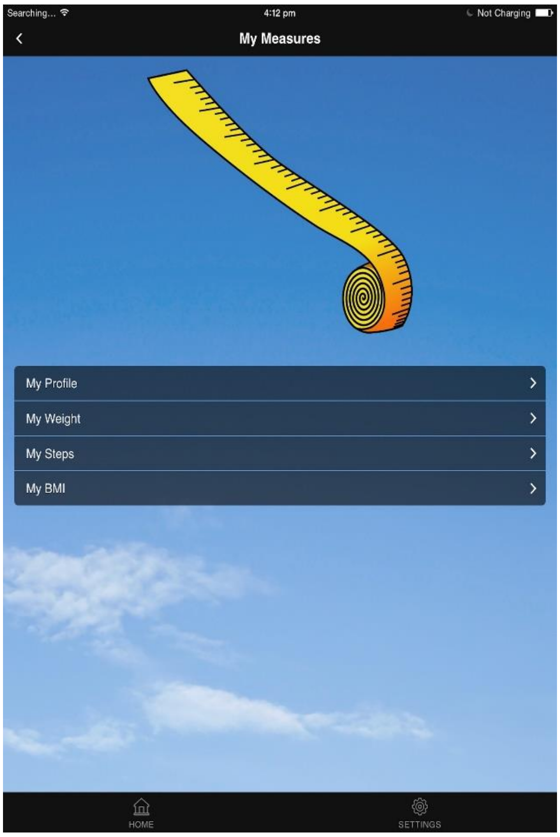

a) Homepage b) My measures

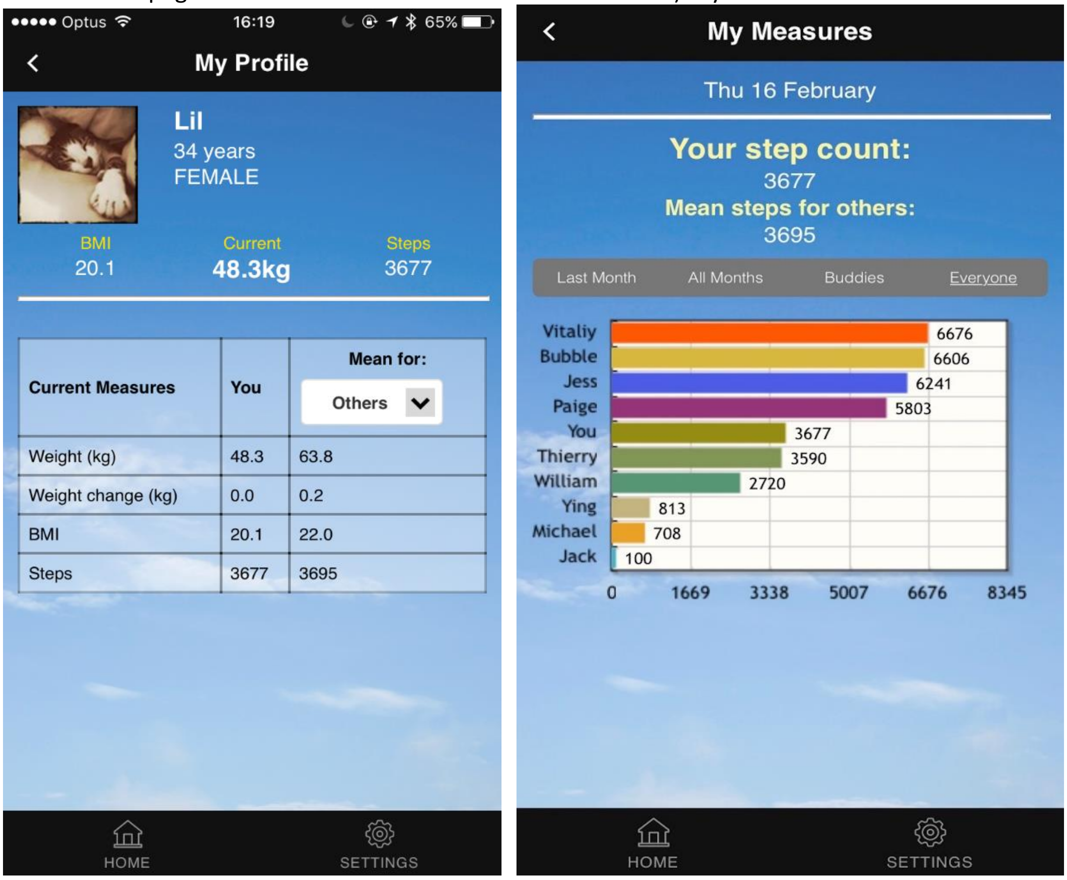

c) Social comparison features
