# Supplementary material for: Efficacy of a Mobile Social Networking Intervention in Promoting Physical Activity: Quasi-Experimental Study
Source: JMIR Mhealth Uhealth. 2019 Mar 28;7(3):e12181. doi: 10.2196/12181 (PMC6458538; doi:10.2196/12181)
Supplement: Multimedia Appendix 2 [file mhealth_v7i3e12181_app2.pdf]

Appendix 2: Responses to individual system usability scale statements

Statement	Raw Mean	Raw SD
**1. I think that I would like to use this application frequently**	2.5	1.1
**2. I found the application unnecessarily complex**	2.7	1.2
**3. I thought the application was easy to use**	3.6	1.2
**4. I think that I would need the support of a technical person to be able to use this application**	1.9	1.1
**5. I found the various functions in this application were well integrated**	3.1	1.0
**6. I thought there was too much inconsistency in this application**	2.8	1.1
**7. I would imagine that most people would learn to use this application very quickly**	3.9	0.9
**8. I found the application very cumbersome to use**	3	1.1
**9. I felt very confident using the application**	3.5	1.0
**10. I needed to learn a lot of things before I could get going with this application**	2.1	1.1

Abbreviation: SD: standard deviation

Note: Response categories vary from 1 (strongly disagree) to 5 (strongly agree). In order to calculate the total system usability score, first the score contributions from each item are summed. For items 1, 3, 5, 7, 9, the score contribution is the scale contribution minus 1. For items 2, 4, 6, 8, 10, the contribution is 5 minus the scale position. Then, the sum of the scores are multiplied by 2.5 to obtain the overall value of system usability. System usability score ranges from 0 to 100.
