# Supplementary material for: Efficacy of a Mobile Social Networking Intervention in Promoting Physical Activity: Quasi-Experimental Study
Source: JMIR Mhealth Uhealth. 2019 Mar 28;7(3):e12181. doi: 10.2196/12181 (PMC6458538; doi:10.2196/12181)
Supplement: Multimedia Appendix 3 [file mhealth_v7i3e12181_app3.pdf]

Appendix 3: Boxplots of the 55 participants' daily step count over 26 study weeks

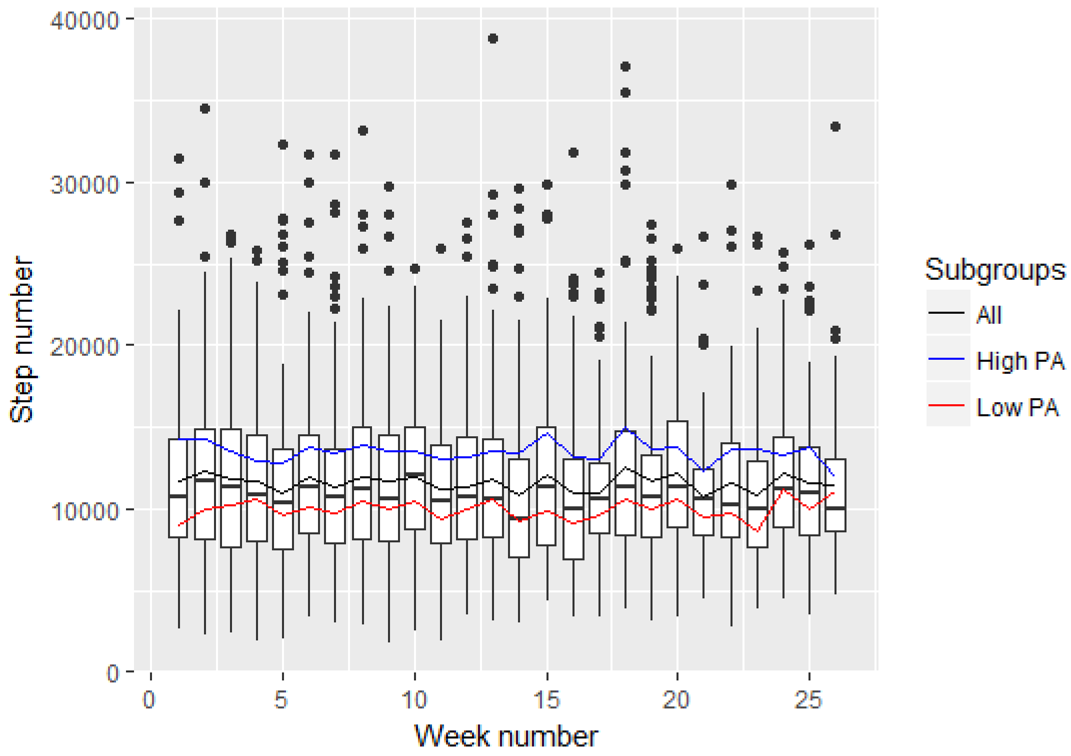

Abbreviations: PA: physical activity**. Notes:** A 'high' physical activity level was defined as having at least 10,000 steps per day on average for the first week, while a 'low' physical activity level was defined as having less than 10,000 steps per day on average during the first week.
