# Supplementary material for: Efficacy of a Mobile Social Networking Intervention in Promoting Physical Activity: Quasi-Experimental Study
Source: JMIR Mhealth Uhealth. 2019 Mar 28;7(3):e12181. doi: 10.2196/12181 (PMC6458538; doi:10.2196/12181)
Supplement: Multimedia Appendix 4 [file mhealth_v7i3e12181_app4.docx]

**Appendix 4. Differences in characteristics between frequent app users and non-frequent app users^a^**

|  | Frequent users  (n=28)  mean (SD) | Non-frequent users  (n=27)  mean (SD) | *P*  (95% CI) |
| --- | --- | --- | --- |
| Baseline weight (kg) | 76.3 (19.3) | 79.9 (25.3) | 0.79^b^  (-13.3, 9.7) |
| Baseline BMI (kg/m^2^) | 26.4 (6.1) | 26.7 (7.5) | 0.86^b^  (-3.0, 3.2) |
| Baseline steps/day | 11534 (3317.5) | 10379 (4424.5) | 0.60^c^  (-1111.5, 2384.2) |
| Pre-post intervention step difference | -117.4 (4472.9) | 168.4 (3533.3) | 0.42^c^  (-2264.1, 1126.1) |
| SUS score^d^ | 65.6 (13.4) | 52.6 (23.5) | 0.04^b^  (0.6, 25.3) |

**Abbreviation**: n: frequency count, SD: standard deviation, *P*: p-value, CI: confidence interval, kg: kilogram, m: metre, SUS: system usability scale; Notes: ^a^The median of frequency (i.e. 1328 times) of app usage is used as a cut-off point to define frequent and non-frequent users, ^b^Assessed using two-sample t-test, ^c^Assessed using Wilcoxon rank sum test, ^d^Only study completers (i.e. participants who returned to the final session) completed the SUS (n=45; 26 frequent users, 19 non-frequent users).
