# Supplementary material for: Efficacy of a Mobile Social Networking Intervention in Promoting Physical Activity: Quasi-Experimental Study
Source: JMIR Mhealth Uhealth. 2019 Mar 28;7(3):e12181. doi: 10.2196/12181 (PMC6458538; doi:10.2196/12181)
Supplement: Multimedia Appendix 5 [file mhealth_v7i3e12181_app5.docx]

**Appendix 5. Differences in characteristics between frequent users and non-frequent users^a^ of the social features in the fit.healthy.me app**

|  | Frequent users  (n=28)  mean (SD) | Non-frequent users  (n=27)  mean (SD) | *P*  95% CI |
| --- | --- | --- | --- |
| Baseline weight (kg) | 72.4 (17.4) | 75.6 (25.5) | 0.06^b^  (-23.3, 0.3) |
| Baseline BMI (kg/m^2^) | 24.9 (5.2) | 28.2 (7.9) | 0.07^b^  (-6.9, 0.3) |
| Baseline steps/day | 11021 (3932.4) | 10911 (3955.4) | 0.77^c^  (-1866.0, 1592.2) |
| Pre-post intervention step difference | -702.8  (4520.4) | 851.3 (3266.6) | 0.25^c^  (-3041.0, 851.5) |

**Abbreviation**: N: frequency count, SD: standard deviation, *P*: p-value, CI: confidence interval, kg: kilogram, m: metre; **Note**: ^a^The median of frequency (i.e. 112 times) of social features usage is used as a cut-off point to define frequent and non-frequent users, ^b^Assessed using two-sample t-test, ^c^Assessed using Wilcoxon rank sum test
